# Consensus-based Sharp-Wave Ripple detection and its application in an alcohol administration model

**DOI:** 10.3389/fnsys.2026.1822457

**Published:** 2026-07-07

**Authors:** Marina Ruelas, Rita Q. Fuentes-Aguilar, Laura Medina-Ceja, Eduardo Morales-Vargas

**Affiliations:** 1 School of Engineering and Sciences, Tecnologico de Monterrey, Zapopan, Jalisco, Mexico; 2 Institute of Advanced Materials for Sustainable Manufacturing, Tecnologico de Monterrey, Zapopan, Jalisco, Mexico; 3 Laboratory of Neurophysiology, Department of Cellular and Molecular Biology, Centro Universitario de Ciencias Biológicas y Agropecuarias (CUCBA), University of Guadalajara, Zapopan, Jalisco, Mexico

**Keywords:** alcohol model, hippocampus, neuroscience, ripple detection, sharp wave ripples

## Abstract

In the hippocampus, slow waves are accompanied by brief population bursts of high-frequency oscillations (150–250 Hz) known as Sharp-Wave Ripples (SWRs), a phenomenon associated with memory consolidation during offline brain states and Non-Rapid Eye Movement (NREM) sleep. Despite the relevance of SWRs, no standardized criterion for their automatic detection has been established. This work introduces a consensus-based algorithm that first identifies sharp waves and then detects ripples occurring within these intervals. Events are designated as true SWRs only when at least two principal methodologies report overlapping detections. Comparative analyses showed that one detector generated more candidate events but with reduced precision, whereas the other was more selective but computationally slower. The consensus strategy improved reliability by emphasizing the concurrence of independent detectors, contributing to efforts toward standardized and reproducible SWR analysis. The algorithm was used within an alcohol administration model to quantify SWR rate, duration, and peak frequency across control, vehicle, and treated groups. Although no significant group-level differences emerged under the short-term exposure protocol, a significant increase in SWR peak frequency was observed in the treated group after the open field test, suggesting the presence of a transient compensation mechanism. These findings shed light on the brain's ability to adapt temporarily to specific behavioral tasks. However, it is essential to emphasize that additional research is crucial to fully understand the long-term implications and associations with alcohol-induced changes in brain structures and SWR generation.

## 1 Introduction

Sharp Wave Ripples (SWRs) are population bursts of high-frequency oscillations (150–250 Hz) lasting from 50 to 100 ms that originate within the interconnected CA2–CA3 subfields of the hippocampus (Cowen et al., 2020). These events occur during ‘off-line' brain states and Non-Rapid Eye Movement (NREM) sleep, periods associated with consummatory actions and memory consolidation. A recent study on hippocampal SWRs reported that ripple activity decreases during reward-related behaviors, such as water licking, even in the absence of overt movement, and increases during immobile periods with reward expectation (Sakairi et al., 2025). These findings suggest that SWRs are more closely related to relative immobility than to consummatory behavior itself, contributing to memory consolidation by transmitting compressed hippocampal representations to distributed neural circuits and have been observed in both humans and rodents (Cowen et al., 2020; Oliva et al., 2020; Buzsáki, 2015). As a result, SWRs have gained increasing attention for their association with the high-fidelity replay of past experiences and the reactivation of hippocampal place-cell sequences that encode spatial and episodic memory.

The detection of SWR remains a challenging task due to the presence of noise, variability in signal characteristics, and the overlap with pathological high-frequency activity. Despite their importance, no universally accepted standard for SWR detection has been established, and even expert annotations may vary across studies. Recent work has emphasized these limitations and the need for standardized and reproducible detection strategies (Liu et al., 2022), motivating the development of approaches that reduce methodological variability.

Despite the growing interest in the automatic identification and analysis of SWRs, only a limited number of procedures have been specifically developed for these tasks. Most existing methods were originally designed to detect high-frequency oscillations (High frequency oscillations (HFOs); 80–500 Hz) during epileptic episodes (Mölle et al., 2004). However, because SWRs occur within a narrower frequency band (120–250 Hz) that lies within the HFO range, methodologies reported for HFO detection can often be adapted to identify glspl SWR. Nonetheless, current approaches present important limitations (Cowen et al., 2020; Oliva et al., 2020; Buzsáki, 2015). In particular, most do not evaluate whether the HFO occurs during a slow wave—an electrophysiological biomarker associated with memory encoding and behavioral planning (Eschenko et al., 2008; Mölle et al., 2006; Ivan et al., 2021; Navarrete et al., 2016). Moreover, despite the development of multiple computational approaches for automatic SWR and high-frequency oscillation detection, there is still no consensus on the optimal combination of features and criteria that define a true SWR event (Kondylis et al., 2014; Burnos et al., 2014; Remakanthakurup Sindhu et al., 2020). SWR are classically described as events composed of a CA3-originated sharp wave and a ripple oscillation in the CA1 pyramidal layer. However, recent studies have shown that these components do not always co-occur, and that ripple events can exhibit substantial variability in amplitude, duration, and anatomical origin (Ramirez-Villegas et al., 2015; Aleman-Zapata et al., 2025). This heterogeneity contributes to the lack of consensus in SWR detection and complicates the establishment of standardized identification criteria.

HFOs detection pipelines can be categorized into three groups: manual, semi-supervised, and fully automated strategies. Manual review by experts is the gold standard, but it is time-consuming and highly subjective to the individual's experience performing the task (Birot et al., 2013; Staba et al., 2002). On the other hand, semi-automated and automated detection systems often follow a sequence of well-defined steps that typically begin with band-pass filtering to extract the frequencies of interest. After bandpassing, the signal is made positive by rectifying or computing the envelope/smoothing. Then, a thresholding detection approach is used to cut off the events; the most used methods threshold an energy (Staba et al., 2002; Zelmann et al., 2010; Charupanit and Lopour, 2017), statistical (Blanco et al., 2010; Shimamoto et al., 2018; Chu et al., 2017; Burnos et al., 2014), or spectral characteristic (Mölle et al., 2004) of the filtered data. Finally, an automated algorithm or semi-supervised validation is performed manually to eliminate false positives. However, despite numerous proposals for automatic HFO detection, there is no established gold standard beyond visual examination. One proposed solution is to implement multiple algorithms across datasets to determine a strategy that performs best with minimal tuning (Birot et al., 2013).

Four ripple detection methodologies were compared within a consensus-based framework in this work. An additional processing stage was incorporated into each conventional approach to ensure that detected ripples occurred within the temporal context of a slow wave, thereby aligning ripple identification with established physiological definitions of SWRs. To assess the contribution of distinct signal characteristics reported in the literature, the analysis included one algorithm based on signal energy thresholding, one relying on spectral features, and two exploiting statistical properties of the signal. Widely adopted ripple detection algorithms, such as *bz*_*F*_*indRipples*.*m* and *FindRipples*.*m* from the Buzsáki and Zugaro laboratories, have established a de facto standard in rodent SWR research. These approaches typically rely on band-pass filtering in the ripple band, estimation of the squared and smoothed signal envelope, and dual-threshold criteria to define event onset, peak, and duration within physiologically grounded limits. In the present study, the signal power–based detector is conceptually related to these methods in that it also relies on amplitude-based characterization of high-frequency activity. However, the implementations differ in normalization procedures, threshold definitions, and event selection criteria. The objective of this work is not to replicate a specific canonical detector, but to evaluate the consistency of SWR identification across diverse detection strategies within a consensus-based framework. This design choice allows us to assess reproducibility across methods while minimizing dependence on the assumptions of any single detection pipeline. Sharp-Wave Ripple detection was evaluated using Intracranial Electroencephalogram (iEEG) recordings obtained from rats subjected to chronic alcohol administration. This experimental model was selected due to well-documented ethanol-induced impairments in hippocampal-dependent cognitive tasks, which are closely associated with SWR-mediated episodic memory consolidation in rodent models.

However, evidence regarding the impact of ethanol on SWRs remains sparse. Preliminary findings in rodents indicate that acute ethanol exposure (1.5 g/kg, i.p.) does not alter hippocampal SWR frequency during rest periods (Miyake et al., 2020). Notably, during a U-track task, ethanol-treated animals exhibited reduced locomotor activity without a concomitant loss of task motivation. This motor impairment likely reflects extra-hippocampal effects, such as cerebellar-mediated ataxia or altered cerebral blood flow secondary to changes in systemic blood pressure. Furthermore, single-unit analysis of place cell firing suggests that ethanol induces a partial reorganization of hippocampal spatial maps. In view of this research gap, we adopted the present consensus- detection framework to evaluate how ethanol modulates the conventional parameters of SWRs.

## 2 Materials and methods

### 2.1 Electrophysiological recordings

A database obtained from an intragastric alcohol model developed in adult male Wistar rats (250–300 g) was used. Twenty-eight Wistar adult male rats were kept under vivarium conditions, that is, they remained in silent rooms with 12 h light/12 h dark cycles (7 a.m. to 7 p.m.), with room temperature (22 − 23°C) and free movement. The animals were fed 85% of their usual diet to keep them motivated for the experiments. The subjects were divided into three experimental groups: a sham group (undergoing surgical implantation and behavioral testing, *n* = 8), a vehicle group (receiving water 0.9%, *n* = 8), and an ethyl alcohol group (EtOH 5 *g*/*kg*/*day*, *n* = 12). The care and performance of the experiments were in accordance with the Health Research Rules, with the approval of the Local Animal Protection Committee with number CINV-C/014/2021, and the provisions of the official standard NOM-062-ZOO-1999. All the experiments were carried out in the Neurophysiology Laboratory of the University of Guadalajara.

The rats were anesthetized with isoflurane in 100% O_2_. They were then positioned in a stereotaxic frame to have lambda and bregma in the same horizontal plane with the incisor bar placed at –3.3 mm. Small holes were drilled to allow the implantation of bipolar tungsten microelectrodes (60 μm diameter, and vertical distance between exposed tips of 1.5 mm). Microelectrodes were implanted in the left posterior hippocampus (AP: –5.0 mm, ML: 5.0 mm, DV: –5.5 mm), right posterior hippocampus (AP: –5.0 mm, ML: –5.0 mm, DV: –5.5 mm), left anterior hippocampus (AP: –3.5 mm, ML: 2.0 mm, DV: –4.0 mm), and right anterior hippocampus (AP: –3.5 mm, ML: –2.0 mm, DV: –4.0 mm). To ensure that the four pairs of bipolar microelectrodes were adequately held, they were placed in a device fastened to the skull with dental acrylic.

Intracranial electrophysiological signals were acquired using pairs of tungsten microelectrodes implanted in anterior and posterior hippocampal regions (left and right hemispheres). Each electrode pair was separated by approximately 500 μm in depth. The impedance of the electrodes ranged between 100 − 300 k at 1 kHz, consistent with typical values for local field potential (LFP) recordings. Signals were amplified, band-limited, and digitized for offline analysis. Given the electrode configuration, recordings provide regional LFP activity but do not allow precise laminar localization of hippocampal subfields (e.g., CA1 pyramidal layer vs. radiatum). Therefore, the spatial origin of detected events, including the distinction between ripple-generating and sharp-wave–generating layers, cannot be resolved with the present setup.

Two different memory experiment environments were tested: an open camp and a T labyrinth. Before six training days, the animals had 3 days of habituation to the open camp and the T labyrinth. After that, eight rats received a water solution intragastrically for 4 days, and 12 rats received intragastrically ethyl alcohol to induce levels of EtOH intoxication at a chronic level. The alcohol was administered through a gastric cannula (stainless steel needle 6*cm* long with an inclination of 7°, Cadence Science, Staunton, VA, USA) which was attached to a syringe containing EtOH administered twice a day for 4 days. The daily dose of EtOH was 5*g*/*kg*/*day*, which generates alcohol levels between 0.25 and 0.30*g%* in the blood of the animals. The remaining eight rats, who were considered the control group, did not receive anything else but food.

Following the alcohol and water treatment, the 28 rats underwent memory experiments for 3 days. iEEG signals were sampled at 5 kHz and registered before and after the experiment every day, which were evaluated to find the ripples. These recordings constituted the input data for the experiments described in the following subsection.

### 2.2 Automatic detection of SWRs

This work implements an ensemble of four non–machine-learning detectors to identify hippocampal ripples within iEEG: (i) an energy-based (RMS) detector (Zelmann et al., 2010), (ii) a signal power (PSD) detector (Mölle et al., 2004), (iii) a gamma–model detector based on iterative background fitting (Blanco et al., 2010), and (iv) a multi-statistical detector that integrates envelope statistics, zero-crossing constraints, and resampling thresholds (Burnos et al., 2014). All methods operate on bandpass-filtered data within the ripple band and differ in how thresholds are defined and validated. In all four implementations, the mean and standard deviation used to define detection thresholds were computed over the entire recording duration. To increase specificity and reduce method-dependent bias, a consensus rule is adopted: only events concurrently detected by at least two methods are accepted as true SWRs. This ensemble strategy balances sensitivity and precision and yields robust estimates of event occurrence, duration, and peak frequency for subsequent statistical analyses across groups and sessions.

#### 2.2.1 Energy-based detector

The first implemented method to identify SWRs was reported by Staba et al. (2002), Figure 1. The iEEG signal was filtered (120–250 Hz, FIR, lower stopband = 60, upper stopband = 500 Hz) using both forward and reverse filtering. Then, ripples were detected: (A) The Root Square Mean (RMS) was computed from the filtered signal using a 3 ms window. (B) Putative ripple events were defined as consecutive RMS values that exceeded 5 SDs (alpha-Energy-based = 5) above the average RMS mean, and these events had to last for more than 6 ms. (C) A single oscillation was defined as a series of occurrences separated by intervals shorter than ten milliseconds.

**Figure 1:**
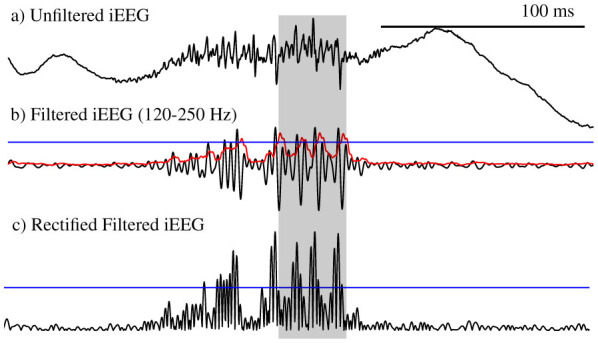
SWR Identification by Energy-based detector. The gray area indicates an SWR, which was identified by the detector. **(a)** Unfiltered signal. **(b)** Filtered iEEG signal (120–250 Hz). The red line represents the Energy-based signal, calculated with a 3 ms window. The blue line depicts the alpha-energy-based threshold, set at 5 SDs above the overall RMS mean. **(c)** Rectified filtered iEEG signal. The blue line indicates 3 SDs above the mean baseline value of the rectified bandpass signal. It is possible to observe more than six peaks above the blue line.

#### 2.2.2 Signal power detector

**Figure 2:**
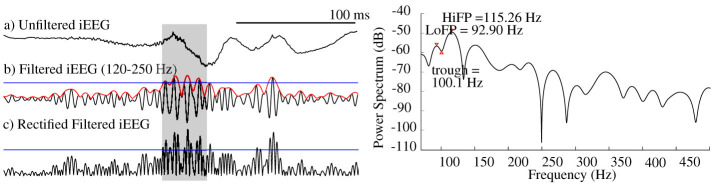
SWR Identification by Signal Power detector. The shaded area indicates an Event of Interest (EoI) identified by the detector. Unfiltered signal. Filtered iEEG signal (120–250 Hz). The red line represents the envelope, calculated with Hilbert transformation. The alpha-Signal Power threshold, set at 3 SDs above the mean of the envelope, is indicated by the blue line. Rectified filtered iEEG signal. The blue line represents 2 SDs above the mean baseline value of the rectified bandpass signal. It is possible to observe more than six peaks above the blue line. Power spectrum of the EoI. The HiFP, trough, and LoFP points are marked with red triangles. The HiFP represents the highest peak in the Signal Power within the frequency range of 70 Hz to 500 Hz.

The second methodology employed for ripple identification was based on the algorithm proposed by Burnos et al. (Figure 2). The signal underwent bandpass filtering (120–250 Hz) using an IIR Cauer filter. The filter specifications included a lower stopband at 110 Hz, an upper stopband at 260 Hz, a lower/upper stopband attenuation of −60 dB, and a maximum passband ripple of 0.5 dB. The signal was filtered both forward and reverse.

The detection of putative ripples followed these steps: (A) The Hilbert transformation was applied to determine the envelope of the band-passed signal. (B) The standard deviation (SD) of the envelope was calculated, and the mean of the envelope plus 3 SDs (alpha-Signal Power = 3) served as a threshold. Whenever the envelope exceeded this threshold, an event was recorded. The interval between the upward and downward crossings of 0.5 times the threshold was used to determine the duration of the event. A potential ripple event was classified if it lasted longer than six milliseconds. (C) Ripple events with inter-event intervals smaller than 10 ms were merged into a single ripple event. Events with a minimum of six peaks in the band-passed signal (corrected above 0 V) deviated by more than 2 SDs from the mean baseline signal were excluded. (D) Subsequently, the period of each putative ripple event [−0.5, +0.5] seconds was transformed into the time-frequency domain and analyzed using the instantaneous power spectra of the Fourier transform representation (PSD, unit: $10log_{10}V^{2}Hz^{-}1$) around the maximum of the envelope. The instantaneous power spectra were computed for all time points of the envelope within the complete width at half maximum above the threshold.

Subsequently, three points were identified from the PSD: (1) A high-frequency peak (HiFP) was selected as the highest peak in the PSD range from 70 Hz to 500 Hz. The methodology proposed by Burnos et al. initially limited the range from 60 Hz to 500 Hz to avoid signal interference from line hum, which was a frequency of 50 Hz in Switzerland, where they conducted their research. However, given that our research took place in Mexico, where the line hum is at 60 Hz, we adjusted the range to 70 Hz to 500 Hz. (2) A trough was determined as the lowest amplitude within the range of 70 Hz to the HiFP. (3) The nearest local maximum below the trough was identified as a low-frequency peak (LoFP).

#### 2.2.3 Gamma model detector

The implementation of the third methodology, proposed by Charupanit and Lopour (2017), Figure 3, involved the following steps for detection. Initially, a bandpass filter (80 − 250 Hz, FIR) was applied to the iEEG recordings, with a lower stopband of 70 Hz and an upper stopband of 260 Hz. The filter had a lower/upper stopband attenuation of −60 dB and was both forward and reverse-filtered. Subsequently, the methodology proceeded as follows: (A) The amplitude of each oscillatory cycle was determined by identifying each peak in the rectified, filtered signal. (B) A histogram was created to visualize the distribution of the peak amplitudes. (C) To approximate the background activity of the channel, a gamma distribution model *f*(*x*) was derived using the generated histogram and Equation 1.


$$\begin{array}{l} f\left(x\right)=\frac{1}{\text{Γ}\left(k\right)\theta^{k}}x^{k-1}e^{\frac{-x}{\theta }},x\in \left(0,\infty \right) \end{array}$$ (1)

**Figure 3:**
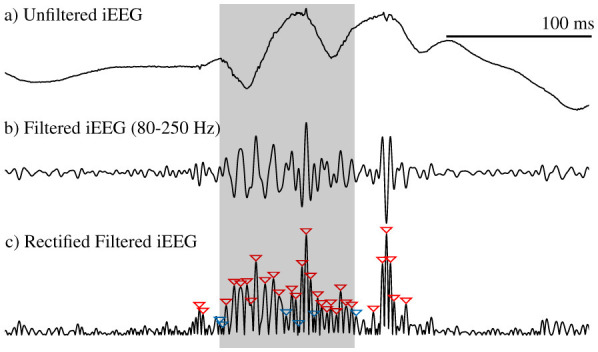
Identification of SWR by Gamma Model detector. The highlighted area represents a SWR event identified by the detector. The process involves the following steps: **(a)** Unfiltered signal, **(b)** Filtered iEEG (80–250 Hz), and **(c)** Rectified filtered iEEG. Red triangles signify peaks surpassing the threshold established during the iteration process, while blue triangles indicate peaks below the threshold within the SWR event.

The gamma probability distribution relies on the shape parameter (*k*) and the scale parameter (θ). These parameters characterized the magnitude of the background activity, resulting in the creation of the estimated probability distribution, denoted as *f*(*x*).

- A *F*(*x*)>1*alpha* (Alpha-Gamma Model = 0.01) limit was established, where *F*(*x*) is the cumulative probability distribution function of *f*(*x*). Peaks with amplitudes greater than the cutoff were removed from the distribution, and *k* and θ were recalculated to obtain a better estimate of the background activity, *f*(*x*).
- With each iteration, the process was repeated with the new estimate of *f*(*x*) until the values of *k* and θ converged and no more peaks were removed. The detection threshold was set based on the final iteration's cutoff value.
- Finally, all peaks with amplitudes greater than the threshold were identified, and SWRs were defined as occurrences with at least five out of six consecutive peaks above the threshold.

#### 2.2.4 Multi-statistical detector

The final implemented ripple identification methodology was proposed by Chu et al. (2017), Figure 4. For the first part of the algorithm, the following steps were followed.

- The data was bandpass filtered in each channel (100–300 Hz, FIR Equiripple filter, order 170, lower stopband = 60 Hz, upper stopband = 350 Hz, lower stopband attenuation = –80 dB, upper stopband attenuation = –40 dB, maximum passband ripple = 0.1 dB, forward and reverse filtered).
- A Hilbert transform was computed to obtain the amplitude envelope.
- An envelope threshold was determined as a percentage of the amplitude envelope values computed for the entire dataset (in this example, Alpha-Multi-Statistical-1 = 85%). All time points where the amplitude envelope exceeded the envelope threshold were recognized. If two of these time points were separated by less than 5 ms, all values between them were declared over the envelope threshold.
- Events with an amplitude over the threshold with a duration of a minimum of twenty milliseconds were marked as putative events.

**Figure 4:**
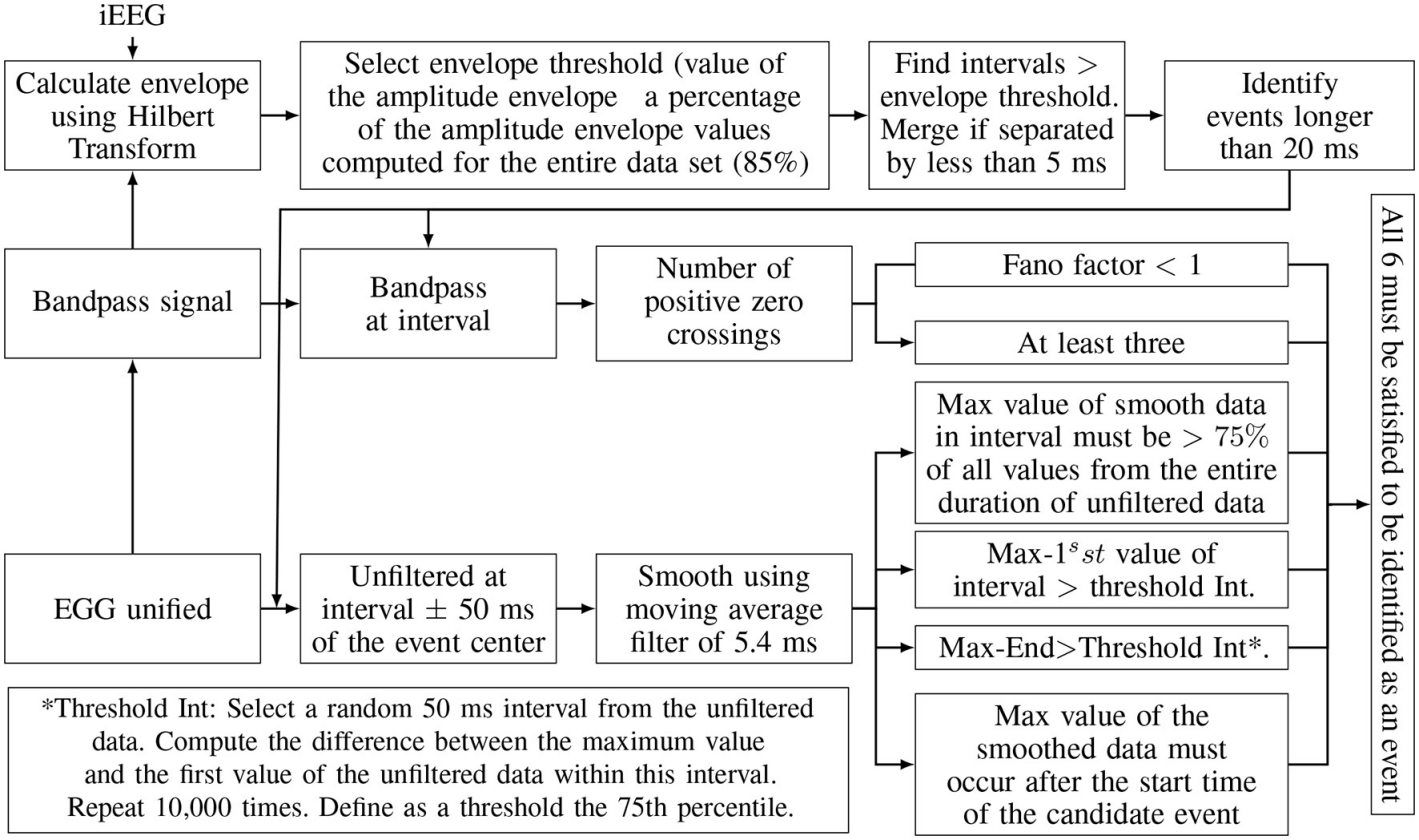
Multi-Statistical detector methodology for ripple identification. Reproduced and modified from Chu et al. (2017).

The second part of the algorithm consisted of testing if the putative ripple events fulfilled the following criteria. 1) Within the bandpass filtered signal of the putative time interval, the positive zero-crossings had to be greater than or equal to three, and the Fano factor computed using the time intervals between zero-crossing had to be less than one. 2) Within the unfiltered data interval associated with the candidate ripple event (smoothed with a moving average filter of length 5.4 ms), four criteria were determined:

- The maximum value of the smoothed data in this interval had to exceed 75% of all values (Apha-Multi-Statistical-2 = 75) from the entire duration of unfiltered data (Max value of smooth data > 75% of all values).
- The maximum value of the smoothed data had to occur after the start time of the candidate ripple event (Max value time < 0).
- The difference between the smoothed unfiltered data's highest value in this interval and the smoothed unfiltered data's value at the beginning and end of the interval was determined (Max-1st value of interval > Threshold Int., and Max-End > Threshold Int.). Both amounts were required to surpass the following threshold value, which was determined by resampling.
- From the whole period of the unfiltered data, a random 50 ms interval was chosen, and the difference between the maximum value and the beginning value of the unfiltered data was computed within this interval.
- The resampling technique was done 10,000 times to generate a distribution of the differences and set the 75th percentile as a threshold (same as Alpha-Multi-Statistical-2).

Recent advances in SWR detection have increasingly incorporated machine learning approaches, including convolutional neural networks (CNNs), recurrent neural networks (RNNs), and transformer-based architectures, as well as methods leveraging high-synchrony multi-unit activity (MUA) as a detection benchmark. These approaches offer the potential to capture complex temporal and spatial features beyond classical amplitude-based criteria. However, they typically require large, well-annotated datasets, substantial computational resources, and may reduce the interpretability of detection decisions. In contrast, the present work focuses on consensus across classical signal-processing–based detectors, which remain widely used and accessible in experimental settings with limited channel configurations. From this perspective, the proposed consensus framework may serve as a complementary approach, particularly for improving reproducibility and for generating robust candidate events that could be used in the future to train or validate machine learning models.

## 3 Methodology

**Figure 5:**
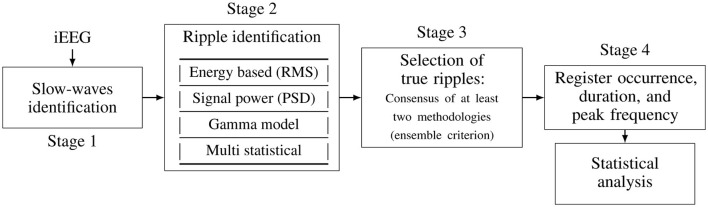
Schematic diagram of the methodological pipeline for SWR detection and analysis. The implemented methodology for ripple identification. First, slow waves were identified, and the time segment in which they occurred was recorded, including an additional two-second interval of [−1*s*, +1*s*]. Then, four different methodologies for ripple identification were implemented within these time segments of slow waves to identify the SWRs. Afterward, ripples detected by at least two ripple identification methodologies were considered valid. Next, the occurrence, duration, and peak frequency of each iEEG recording were computed. These metrics allowed for a comprehensive assessment of these parameters for each data set. A statistical analysis was conducted to evaluate the differences among and within groups.

This section describes the methodological workflow followed to identify and quantify SWRs in iEEG recordings (Figure 5). In this study, low-frequency activity in the 0.5−−4 Hz range was used as an operational reference to constrain ripple detection. We note that this activity may reflect a combination of hippocampal sharp waves and volume-conducted slow oscillations, and that its precise anatomical origin cannot be determined with the present recording configuration. Importantly, this constraint was used as a signal-processing criterion rather than as a strict physiological definition of SWR events. Previous studies have shown that ripple events can occur independently of prominent slow deflections (Ramirez-Villegas et al., 2015), and therefore the coupling criterion adopted here may bias detection toward a subset of events. First, slow waves were detected channel by channel using the criteria proposed by Riedner and Valderrama, after preprocessing with Chebyshev filters (low-pass filter at 30 Hz and a bandpass filter within the range of 0.5 − 4.0*Hz*). Each slow-wave event defined an extended analysis window from −1 s to +1 s around the detected slow wave to estimate the baseline. Second, within these windows, four classical ripple detectors were implemented (Staba/RMS, Burnos/PSD, Charupanit & Lopour/Gamma Model, and Chu/Multi-Statistical), adjusted to the passband of 120 − 250*Hz*. Third, a conservative ensemble was constructed: an event was considered a *true SWR* only if it was marked by at least two detectors with temporal overlap; the onset and offset were defined by the earliest and latest detected time points, respectively. Fourth, for each detected *SWR*, peak frequency (via fft of the filtered segment) and duration were computed as event-level features. The occurrence rate, in contrast, was calculated at the segment level as the number of SWRs per minute within each recording interval. Finally, to assess statistically significant differences across the experimental conditions, sham, vehicle control, and ethanol groups, a one-way Analysis of Variance (ANOVA) was employed. This approach was selected to evaluate the null hypothesis that the mean values of the dependent variable are equal across all three independent cohorts. By utilizing a single global test prior to *post-hoc* comparisons, we minimized the inflation of Type I error associated with multiple pairwise comparisons, thereby ensuring the statistical populations were compared with appropriate rigor, after verifying the normality assumption and applying a logarithmic transformation when necessary (*p* < 0.05).

### 3.1 Stage 1: slow-wave detection

**Figure 6:**
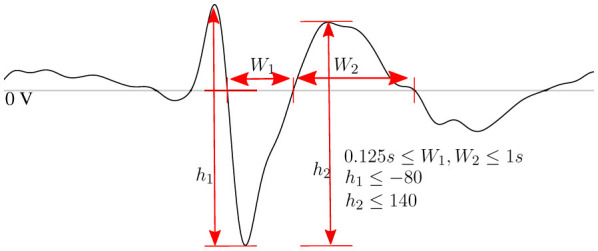
Criteria for slow waves detection.

First, slow waves were identified in each channel using the methodology suggested by Riedner and Valderrama, which will be discussed later. Second, the time segment in which the slow waves were identified was recorded, and an additional interval of [−−1*s*, +1*s*] was added to extend the window. Slow wave detection was carried out using the methodologies proposed by Riedner and Valderrama in Matlab (Riedner et al., 2007; Valderrama et al., 2012). The following steps were followed for slow wave identification. First, using a Chebyshev Type II filter, the signal was subjected to a low-pass filter at 30 Hz and a bandpass filter within the range of 0.5–4.0 Hz (with a lower stopband at 0.1 Hz and an upper stopband at 10 Hz). Second, the following requirements had to be met to be classified as a slow wave: (1) The waveform should exhibit a negative peak of equal to or less than –80 μV with only one primary peak between two successive zero-crossings, spaced between 0.125 and 1 s. Additionally, any other negative peaks should be less than fifty percent of the magnitude of the primary peak; (2) a positive wave, either subsequent or antecedent, should appear between two consecutive zero-crossings, with a time interval ranging from 0.125 to 1 s; (3) The peak-to-peak amplitude of the negative-to-positive (or positive-to-negative) waveform should exceed 140 μV. These criteria are depicted in Figure 6.

A preliminary test was conducted to evaluate the accuracy of this detector. An expert analyzed thirty-five short iEEG segments each lasting from 1.6 to 2.2 seconds, and the algorithm's results were compared to the expert's findings.

### 3.2 Stage 2: ripple detection

After identifying slow waves, we employed four distinct automated methods to detect ripples within the time segments corresponding to them. In other words, we assessed the unfiltered intervals defined between the beginning and end of the identified slow waves, extending these intervals by an additional two seconds. This extension was necessary because the algorithms detect ripples by estimating a baseline and identifying higher oscillations within it. Restricting the analysis solely to the ripple window would have hindered our ability to detect these oscillations accurately.

The evaluated methodologies were proposed by four different papers, Zelmann et al. (2010), Mölle et al. (2004), Blanco et al. (2010), and Burnos et al. (2014), and are briefly described in the following subsections. These algorithms use different characteristics of the signal to determine the threshold to pinpoint SWRs. The methodology proposed by Blanco et al. (2010) uses the energy of the filtered signal and it will be referred to as the RMS detector. The algorithm by Mölle et al. (2004) will be cited as the PSD detector because it works with the power spectral features of the signal to detect the SWRs. Finally, the remaining two methodologies utilize statistical characteristics of the signal. The methodology by Blanco et al. (2010) will be named Gamma Model detector, and the one by Burnos et al. (2014), Multi-Statistical detector.

The four methodologies employed different bandpass filters for the signal. Initially, only the Signal Power and Gamma Model detectors applied forward and reverse filtering to mitigate offset. However, each detector underwent forward and reverse filtering to benchmark the start and end of events in our study.

Furthermore, the original papers used Energy-based and Signal Power detectors to identify HFOs. However, our implemented methodology focused solely on ripples; consequently, we adjusted the passband frequencies to 120 – 250 Hz.

### 3.3 Stage 3: ensemble for true SWRs

**Figure 7:**
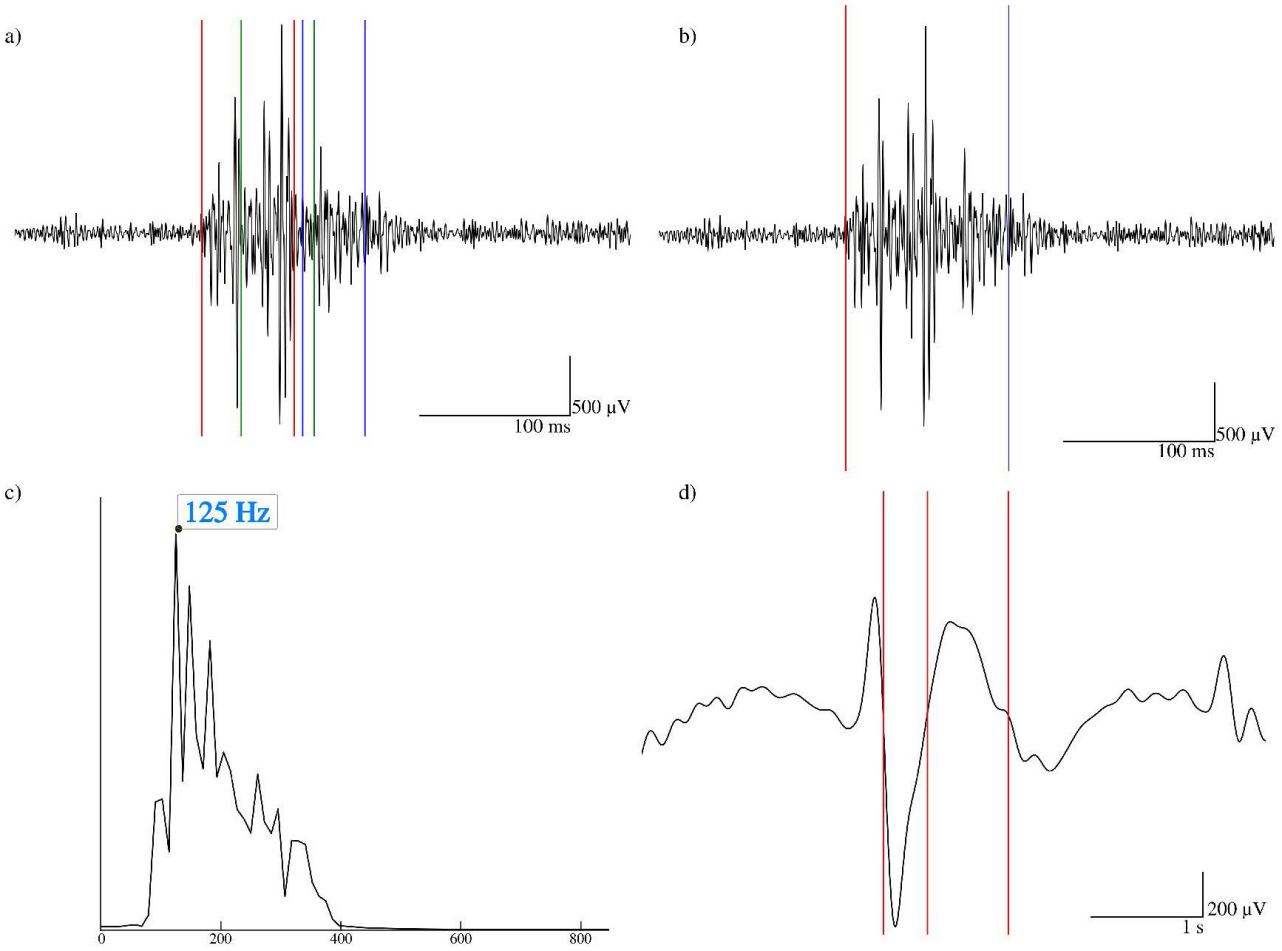
The selection of valid ripple events is based on the overlapping results of different methodologies. **(a)** In this example, three identification methodologies had overlapping signals for a single SWR event (red: Energy-based detector; blue: green: Signal Power detector; Gamma model detector). **(b)** The overall event is considered a true ripple. The beginning and end of the event are determined by selecting the smallest (red line) and greatest (blue line) time markers identified by the three methodologies. The duration of the event is the subtraction between the greatest and the smallest time marks. **(c)** Peak Frequency selection. A Fourier transform was performed during the event selection, and the maximum peak was chosen as the peak frequency. **(d)** Example of an identified slow wave.

Once the SWRs were identified using the four methodologies, they were considered valid only if detected by more than one ripple identification methodology. This means any time interval overlap determined by two or more methodologies was deemed a true SWR (see Figure 7a). The smallest mark determined the event's beginning, while the event's end was considered by the greatest mark, as illustrated in Figure 7b.

### 3.4 Stage 4: quantification of SWR

Upon confirming the authenticity of a SWR event, the peak frequency was determined by applying a bandpass filter to the raw data of the recording, mirroring the parameters of the Energy-based detector. The ensuing steps unfolded as follows:

- A Fourier transformation was performed to the filtered signal.
- The maximum peak was identified. The resulting peak frequency of the event was registered (Figure 7c).

Furthermore, the occurrence of SWR events was calculated by determining the ratio between the total number of true SWRs and the duration of the iEEG register, resulting in a SWR/minute ratio. Additionally, the duration of each SWR event was calculated by subtracting the starting time from the ending time of the SWR event (Figures 7b, d).

A straightforward ANOVA model was employed to compare SWR/minute, duration, and peak frequency among three groups of rats: the experimental group that received alcohol, the vehicle group that received water, and the control group with no intragastric treatment. The evaluation was conducted within groups to compare the progress over 3 days (1–3 days; before-after test) and among groups to compare each day and the overall average of the 3 days before and after the behavioral evaluation. Prior to the evaluation with parametric tests, all results were assessed for normality, and logarithmic transformation was applied to test non-normal distributions. Statistical significance was set for all analyses at *p* < 0.05.

### 3.5 Consensus-based SWR identification framework

To improve the robustness and reproducibility of SWR detection, we implemented a consensus-based framework that integrates the outputs of multiple detection algorithms. Rather than relying on a single method, which may be sensitive to specific parameter choices or signal characteristics, this approach defines candidate SWR events based on agreement across independent detectors.

In this study, each detection algorithm was applied independently to the same preprocessed signal, generating a set of candidate ripple events characterized by their temporal intervals. An event was classified as a “consensus SWR” when at least two detectors reported temporally overlapping detections within a predefined tolerance window. Temporal overlap was defined as the intersection between event intervals exceeding a minimum duration threshold, ensuring that detections correspond to the same underlying electrophysiological event.

The selection of a minimum of two detectors reflects a pragmatic balance between sensitivity and specificity. Individual detectors differ in filtering strategies, threshold definitions, and feature representations, which may lead to variability in detected events. Requiring agreement across at least two methods reduces dependence on detector-specific biases and decreases the likelihood of false positives arising from noise or methodological artifacts. At the same time, this threshold preserves a sufficient number of events for downstream quantitative analysis. Increasing the consensus requirement, for example by requiring three or more detectors, would be expected to improve specificity by emphasizing events consistently identified across multiple methods, but at the cost of reduced sensitivity and potential exclusion of lower-amplitude or shorter-duration events. Conversely, reliance on a single detector would maximize sensitivity but reduce robustness. Therefore, the selected threshold should be interpreted as a practical balance rather than an optimal or universal criterion, this consensus criterion is not intended to define ground truth for SWR detection. Instead, it provides an internal reproducibility framework in which agreement across independent detection pipelines is used as a proxy for event reliability. Although overlap between detectors may still reflect shared methodological biases, the use of multiple detectors with different signal-processing assumptions reduces the influence of any single algorithm on the final set of detected events.

Importantly, the consensus criterion is not intended to define a ground truth for SWR detection. Instead, it provides a reproducibility-oriented framework in which agreement across independent detection pipelines serves as a proxy for event reliability. While overlap between detectors may still reflect shared biases, the inclusion of methods with distinct signal-processing strategies reduces the likelihood that all detections arise from a common artifact. From this perspective, the proposed framework emphasizes consistency across methods rather than adherence to a single detection paradigm.

This approach is particularly relevant in the absence of standardized detection criteria and in contexts where manual annotation is limited or subject to inter-rater variability. Furthermore, the consensus framework may serve as a complementary strategy to emerging machine learning–based approaches, for example by providing robust candidate events for training or benchmarking purposes.

## 4 Experiments and results

### 4.1 Identification of SWR

A total of 168 iEEG recordings from 28 rats were subjected to analysis for the presence of SWRs using four different ripple identification methodologies. Among these recordings, the Signal Power detector identified the highest number of events, totaling 527,890 SWRs, followed by the Gamma model detector, which yielded 259,358 SWRs. The Energy-based detector registered 52,825 events, while the Multi-Statistic detector identified the fewest putative SWRs, amounting to 5,046. However, the divergence extended beyond the number of SWRs detected. The computational time varied significantly among the methodologies. The Multi-Statistic detector required the longest computation time of the four detectors. Compared to the Energy-based detector, the Multi-statistic detector took an average of 31.27 times longer to compute the algorithm. The Signal Power detector took an average of 13.55 times more, and the Gamma Model detector took an average of 2.79 times more than the Energy-based detector.

**Figure 8:**
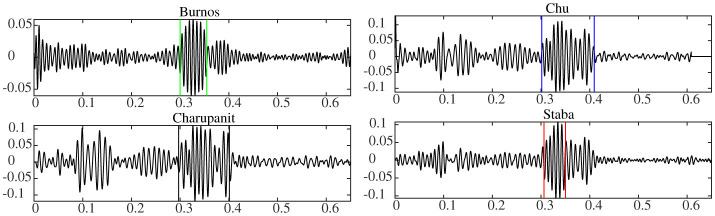
The graphs illustrate SWR events identified using the four different methodologies. The iEEG signal was filtered using the Energy-based (Staba) detector filter. The X-axis represents time (seconds), and the Y-axis represents voltage (μ*V*). Signal Power (Burnos) and Gamma Model (Charupanit) detectors did not overlap in this example; however, the event was identified by the overlapping Gamma Model, Multi-statistical (Chu), and Energy-based detectors. This event was identified by the overlapping of all four detectors.

**Figure 9:**
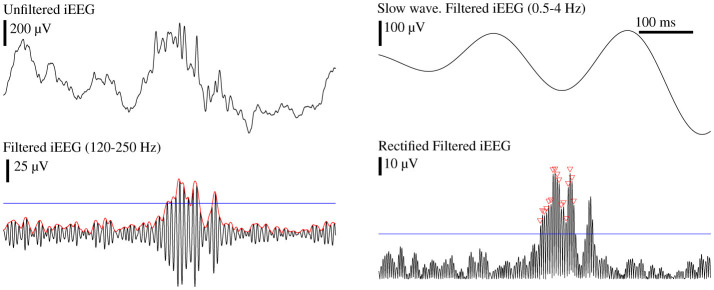
The images display an example of a true positive SWR identified by the Energy-based detector. The SWR is delimited in gray. Unfiltered signal. Slow wave. Signal filtered (0.5–4 Hz). Filtered iEEG (120–250 Hz). Filtered and rectified iEEG. Red triangles indicate peaks that surpass the threshold. In c and d, the horizontal blue line represents the 5 SDs and 3 SDs thresholds, respectively.

The number of SWRs detected and the computation time were not the only differences among the four methodologies for the automatic detection of ripples. Each methodology employed a distinct criterion to determine the initiation and termination points of ripple events, leading to discrepancies in the duration of these events. This variation can be observed in Figure 8, where the same SWRs event is delimited by the four methodologies but with different starting and ending points. Furthermore, the disparity in the filter used is evident in these figures, as the shape of the filtered signal varies for each detector. Figure 9 represents an example of an identified SWR by the Energy-based detector. The event was later confirmed as a positive event since it overlapped with the results from the other three methodologies. In this example, it was observed that nine peaks exceeded 3 SDs in the Rectified Filtered iEEG.

**Figure 10:**
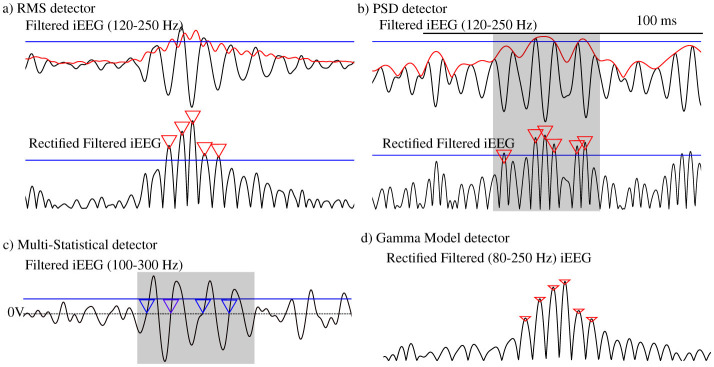
An example of a false negative event identified by the Energy-based detector. **(a)** Energy-based detector. **(b)** Multi-Statistical detector. **(c)** Gamma Model detector. **(d)** Signal Power detector. Each detector is represented with a horizontal blue line indicating the respective threshold, and red triangles mark peaks exceeding the threshold. The SWR is delimited in gray. The second graph of d) red triangles indicates the three identified points (HiFP, trough, and LoFP) that satisfy the criteria set by the Signal Power detector for SWR identification.

On the contrary, Figure 10 shows an example of a SWR rejected by the Energy-based detector but identified by the other three detectors. This occurrence represents a false negative event by the Energy-based detector, which was discarded due to having only five peaks above 3 SDs in the Rectified Filtered iEEG. Additionally, Figure 10 depicts the criteria satisfied by the other three methodologies, while Table 1 provides the additional criteria utilized by the Multi-Statistical detector for this SWR event.

**Table 1:** Criteria of the Multi-Statistical detector for a SWR event rejected by the Energy-based detector.

Evaluated criteria	Result
Number of positive zero-crossings >=3	4
Fano factor < 1	0.5135
Max value of smooth data > 75% of all values	0.4730 > 0.2347
Max-1st value of interval > Threshold Int.	0.3533 > 0.3409
Max-End>Threshold Int.	0.5862 > 0.3409
Max value time < 0	–0.0492

#### 4.1.1 Selection of true ripple events

The number of overlapping events, identified as true SWRs events, showed variation among the evaluated pairs of methodologies (Table 2). Among these pairs, the Signal Power and Gamma Model detectors exhibited the highest overlapping SWR events, totaling 62,600 SWRs. Conversely, the Energy-based and Multi-Statistical detectors had the fewest overlapping events, with only 249 SWRs in common. The total number of events accepted as true events was 98,331. Out of these events, the detector that identified the larger quantity of true ripples was the Gamma Model, which detected 94.19% of the events. The Multi-Statistical detector only identified 2.03% of the events accepted as true.

**Table 2:** Overlapping of SWRs by a pair of methodologies.

Method	Energy-based	Signal power	Gamma model	Multi-statistical
Energy-based	–	22,289	34,952	249
Signal power	22,289	–	62,600	392
Gamma model	34,952	62,600	–	1,632
Multi-statistical	249	392	1,632	–

To conclude, the PSD detector lowered the event-selection threshold from 5 SDs to 3 SDs of the overall mean of the envelope, as originally reported by the RMS detector (Mölle et al., 2004). In our implementation, the PSD detector found more than 9.9 times the number of SWRs than the RMS detector. Also, only 42.19% of the events detected by the PSD detector overlapped with the ones detected by the RMS detector. This means that lowering the number of SDs in the initial step does augment the sensibility of the algorithm, as it was intended, but the second part fails to match the specificity of the RMS detector. Furthermore, the computation time of the PSD detector was 13.55 times longer than the RMS detector. Also, this detector identified 79.25% of the events that were accepted as true ripples by our implementation.

The Gamma model detector used an iterative process based on modeling the rectified peaks of the signal to the gamma distribution (Blanco et al., 2010). They focused on improving the sensibility of the RMS detector, and they reported 24% higher sensitivity when they did a cross-validation test. In our research, the Gamma Model detected almost five times more events than the RMS detector but was the detector with the highest matching percentage with 66% of their events overlapping. Also, implementation is twice the time of the RMS detector, which makes this detector the closest in execution time to the RMS detector. Furthermore, this detector had the highest percentage of identified ripples out of the true events (94.19%).

The last computed methodology was the Multi-Statistical detector (Burnos et al., 2014). This methodology has a lower sensibility as it uses more parameters than the other methodologies to eliminate false positive SWRs events. This methodology reported a sensibility of 52.25% when they compared their results to a visual examination performed by an expert. In our research, the Multi-Statistical detector identified ten times fewer events than the RMS detector, which resulted in an overlapping of barely 0.47% of events. Additionally, the Multi-Statistical detector was the one with the longest computational time with over 31 times the running time of the RMS detector. Also, this detector only identified 2.03% of the events accepted as true by our proposed detector.

Overall, these methodologies did not report if they altered the filter specifications of the RMS detector when they implemented them to benchmark their algorithms. As it may be observed in Figures 1, 2, the filters of all four methodologies resulted in different shapes of the signal. This questions the use of rigid templates to identify the ripples, such as the one used in the PSD detector, where events with less than six oscillations above the threshold were rejected. Perhaps the use of different filters should be evaluated to establish different templates for the detection of these events. Additionally, in future work, all four methodologies could be implemented with the same filter to evaluate if the number of SWRs detected changes with different filter specifications.

Furthermore, although the filter offset was corrected by filtering the signal both forward and reversed, the time interval at which the events are detected was different depending on the filter (see Figure 8). This is a limitation to our study because we considered as true ripples the events that overlapped.

### 4.2 Calculation of SWR/minute, duration, and peak frequency in a model of alcohol administration

An analysis was performed to evaluate whether an alcohol administration regimen comparable to chronic exposure produces measurable alterations in hippocampal SWR activity. Specifically, SWR occurrence rate, duration, and peak frequency were analyzed to assess potential functional changes associated with alcohol exposure. These parameters represent fundamental descriptors of SWR activity and are closely associated with hippocampal communication and memory-related processes. Quantifying their behavior provides a means to assess potential functional alterations induced by alcohol exposure. This evaluation is motivated by extensive evidence indicating that SWRs reflect synchronized communication between the hippocampus and cortical and subcortical structures and play a critical role in memory consolidation, particularly during NREM sleep (Born, 2010; Mölle and Born, 2011; Todorova and Zugaro, 2020; Skelin et al., 2019). In parallel, chronic alcohol consumption has been consistently associated with impairments in cognitive functions, including memory, suggesting that alcohol-induced alterations in SWR dynamics could provide a mechanistic link between neurophysiological disruption and behavioral deficits (Abrahao et al., 2017; Miyake et al., 2020; Harvey et al., 2019; Bird et al., 2018).

**Figure 11:**
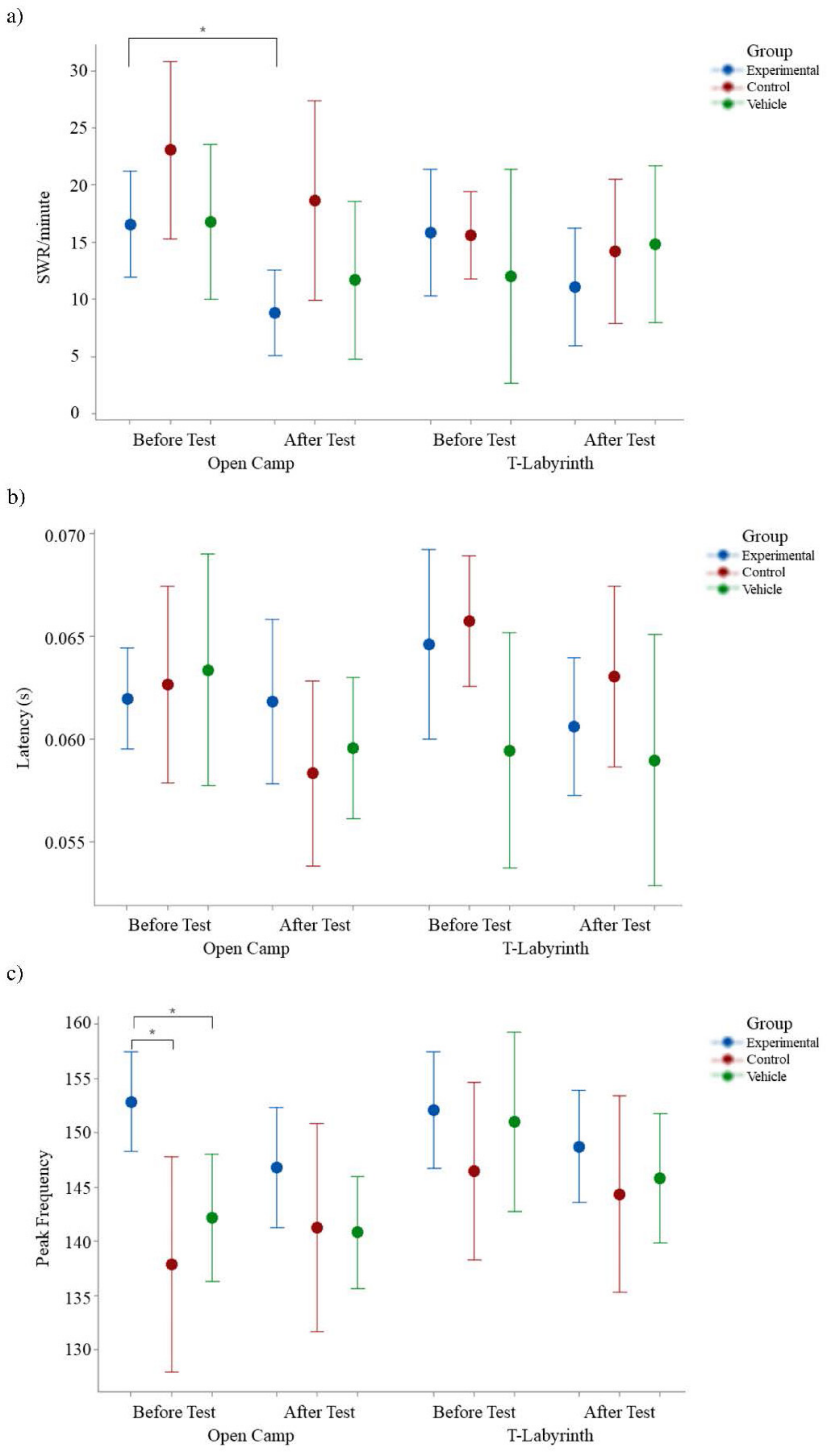
Statistical comparison between different animal groups on open field and T-labyrinth. Each graph shows the mean ± standard deviation (*p < 0.01). **(a)** SWR/min parameter. The evaluation resulted in a significant difference between SWR/min before and after the behavioral assessment when considering the 3 days of the experiment together for the experimental group. **(b)** Duration parameter. **(c)** Peak Frequency parameter.

To address this question, a statistical analysis of SWR occurrence rate, duration, and peak frequency was conducted using data from sham, vehicle, and alcohol groups. These metrics were computed from automatically detected SWR events with the proposed consensus-based method and compared across groups and behavioral conditions (Figures 11a, b).

The mean duration of all recorded SWR events was 61 ms. The shortest mean duration (49 ms) was observed in an iEEG recording from a control-group rat during the open field test on the second day after the behavioral examination. In contrast, the longest mean duration (86 ms) was observed in an experimental-group rat during the T-labyrinth task on the first day prior to the memory test. ANOVA showed no statistically significant differences in SWR duration when comparing recordings obtained before and after the behavioral test in the experimental group for either the open field (*p* = 0.949) or the T-labyrinth task (*p* = 0.147). Additionally, no significant differences were observed when comparing sham, vehicle, and alcohol goups across the 3 days using pre-behavioral recordings for the open field (*p* = 0.866) or the T-labyrinth task (*p* = 0.140).

The average peak frequency across the 168 recordings was 146.5 Hz. The highest average peak frequency (181.19 Hz) was observed in an iEEG recording from an experimental-group rat on the third day following the T-labyrinth task. In contrast, the lowest peak frequency (115.72 Hz) was observed in an iEEG recording from a control-group rat during the open field test on the first day. ANOVA revealed a statistically significant difference in peak frequency among the alcohol, sham, and vehicle groups when recordings from the 3 days prior to the open field test were aggregated (*p* = 0.003; Figure 11c). No significant difference was observed for the T-labyrinth task under the same conditions (*p* = 0.451; Figure 11c).

The analysis did not reveal significant group-level differences among the control, vehicle, and alcohol-treated groups, failing to support the initial hypothesis. The absence of significant effects may be attributable to the alcohol administration model employed in this study. Although the animals reached blood alcohol concentrations between 0.25 and 0.30 g%, consistent with a chronic exposure paradigm, the intragastric administration of alcohol at a dose of 5 g/kg/day, delivered in two daily doses over four consecutive days, may not have been sufficient to induce detectable alterations in brain structures involved in memory consolidation or in the electrophysiological mechanisms that underlie SWR generation. It is possible that the brain plasticity process associated with alcoholism, which affects brain structures relevant for SWR activity at cellular and molecular levels, requires several months in a stable pathological state to reflect alterations in communication between the hippocampus and cortical and subcortical structures, as seen in animal models.

These results are related to differences found in the number of successful trials and task execution. Specifically, during the evaluation period, there were significant differences in the number of successful trials among the sham group (31.17 ± 0.54) and the vehicle group (27.17 ± 1.4) compared to the alcohol-treated group (23.50 ± 0.77) [*F*(3, 18) = 187, *p* ≤ 0.0001]. Additionally, significant differences were observed in task execution time between the vehicle group (7.7 ± 0.52 s) and the alcohol group (13.33 ± 0.37 s) [*F*(3, 18) = 187, *p* ≤ 0.0001]. These results suggest that alcohol exposure had measurable effects on behavioral performance, which may relate to the observed modulation of SWR features.

Finally, with respect to the significant results within the alcohol group after the execution of the open field test and the increase in the peak frequency of the SWR before the open field test with respect to the other vehicle and sham groups. These results may be due to a transient compensatory mechanism to properly execute the test, given that there are still no permanent changes in brain structures that affect the generation of SWR.

## 5 Discussion

A central aspect of this work is the use of a consensus criterion, whereby events are considered SWRs when detected by at least two independent methods. It is important to emphasize that this criterion is not intended to define a ground truth for SWR detection. Instead, it provides an operational framework aimed at increasing detection reliability in the absence of universally accepted standards. Given that even expert manual annotations may vary across observers and experimental conditions, particularly in complex datasets, agreement across multiple automated detectors can serve as a practical proxy for identifying consistent signal features. In this sense, the consensus approach prioritizes reproducibility and robustness over sensitivity to individual detection strategies. The results demonstrate that different detection algorithms yield substantially different sets of candidate events, reflecting variations in sensitivity, selectivity, and signal-processing assumptions. Some methods tend to identify a larger number of events with lower specificity, while others are more conservative but computationally demanding. By integrating these approaches, the consensus framework reduces the influence of individual detector biases and yields a subset of events that are consistently identified across methods. Importantly, the final SWR durations obtained after consensus are consistent with previously reported physiological ranges, even though shorter temporal thresholds were used during initial candidate detection stages. This highlights the importance of distinguishing between preliminary event detection and the final characterization of SWRs.

An important consideration in SWR research is the growing evidence of ripple heterogeneity. While classical definitions describe SWRs as events composed of a CA3-driven sharp wave and a CA1 ripple, recent studies indicate that ripple activity spans a continuum of waveform characteristics and may arise from multiple anatomical generators. Variations in frequency, amplitude, and spatial origin have been linked to distinct synaptic inputs and circuit mechanisms, suggesting that SWRs cannot be fully captured by a single canonical definition (Ramirez-Villegas et al., 2015; Fernández-Ruiz et al., 2019; Aleman-Zapata et al., 2025; Sebastian et al., 2023; Aleman-Zapata et al., 2022; Castelli et al., 2025; Paleologos et al., 2025). Furthermore, distinct subclasses of ripple events with different physiological and functional properties have been described, emphasizing the need for caution when interpreting detected events as a homogeneous phenomenon. In this context, the present approach does not attempt to resolve ripple subtypes, and the detected events should be interpreted as a subset of SWR-related activity defined by the selected detection criteria.

The choice of detection methods is another relevant aspect of this study. The algorithms evaluated were originally developed in the context of high-frequency oscillations (HFOs), particularly in clinical and epileptic settings. In this work, these methods were adapted and parameterized to target physiological ripple activity in the 120–250 Hz range. While other detectors have been specifically developed for rodent SWRs, the selected methods provide diverse signal-processing strategies that enable systematic comparison and integration. The goal of this study is not to establish a definitive benchmark against all existing approaches, but to explore whether combining different methodologies can improve detection consistency. Emerging machine learning–based methods offer promising alternatives; however, they typically require large annotated datasets and introduce additional challenges related to interpretability and generalization.

The application of the consensus-based detection framework to an alcohol administration model serves as a use-case example to illustrate how methodological choices may influence the interpretation of SWR dynamics. While no consistent group-level differences were observed across all measured parameters, condition-specific changes in SWR peak frequency were detected following behavioral testing. These findings suggest that SWR features may exhibit transient modulation under specific experimental conditions. However, these results should be interpreted with caution. The present study does not establish a causal relationship between alcohol exposure and SWR dynamics, and the interpretation of these effects remains limited by the scope of the experimental design. However, these results may be interpreted as follows: the elevated peak frequency of SWRs observed in ethanol-treated animals prior to task execution correlates with impaired performance, specifically characterized by a reduced success rate and increased time of execution. These findings likely arise from ethanol-induced disruptions in the excitatory/inhibitory (E/I) balance within hippocampal CA1 pyramidal neurons. Specifically, ethanol is known to potentiate a subset of GABAergic interneurons via GABA-A receptors (Ariwodola and Weiner, 2004; Wu et al., 2005) while concurrently inhibiting glutamatergic NMDA receptors (Schummers and Browning, 2001). Given that SWR generation is primarily driven by interneuronal activity (Yang and Sun, 2025), such modulation likely induces temporal shifts in SWR peak frequency. This hypothesis is supported by evidence that ethanol increases the frequency of miniature inhibitory postsynaptic currents (mIPSCs) and enhances tonic GABAergic currents in the CA1 region (Weiner and Valenzuela, 2006; Aleman-Zapata et al., 2022). Furthermore, Wallner et al. (2003) demonstrated that ethanol primarily targets GABA-A receptors containing alpha 4 and delta subunits, which are prevalent in the dentate gyrus. To date, literature regarding the effects of alcohol on SWRs remains limited; notably, Miyake et al. (2020) reported that acute systemic administration of ethanol (1.5*g*/*kg*, i.p.) does not alter hippocampal SWR frequency during periods of behavioral quiescence.

Several limitations of this study should be acknowledged. First, the spatial resolution of the recordings does not allow precise localization of SWR components across hippocampal layers, nor does it permit the characterization of ripple propagation or subtype classification. The recordings were obtained using pairs of tungsten microelectrodes, which provide regional but not laminar resolution. Second, the consensus-based detection approach is not validated against a definitive ground truth, as no universally accepted standard for SWR identification currently exists. Therefore, the results should be interpreted in terms of relative detection consistency rather than absolute accuracy. Third, the analysis focuses on wakefulness and task-related conditions, and does not include SWRs occurring during NREM sleep, which represent a distinct and extensively studied physiological context. Fourth, a primary limitation of this work is the absence of expert-validated SWR detection and the lack of benchmarking against standard software like RippleLab. Fourth, while the use of multiple detectors increases robustness, it also introduces additional complexity in parameter selection, highlighting the need for future work on automated or adaptive thresholding strategies. Finally, the use of an arbitrary consensus threshold involving only two detectors for SWR validation represents a limitation of the current methodology.

In summary, this study presents a consensus-based framework for SWR detection that emphasizes robustness and reproducibility by integrating multiple detection methodologies. By addressing variability across detectors and providing a systematic comparison, the proposed approach contributes to ongoing efforts toward standardization in SWR analysis. Future work should extend this framework to high-density recordings, incorporate validation against expert annotations or curated datasets, and explore its integration with emerging machine learning techniques.

## 6 Conclusions

A comprehensive evaluation of four ripple identification methodologies was conducted to identify SWRs. The analysis revealed several important directions for future research and potential enhancements in SWR identification. As a crucial next step, it is essential to benchmark the implemented filters used in each methodology. Some methodologies rely on the signal's shape (e.g., the Multi-Statistical detector requiring events to have at least three positive zero-crossings), which can vary depending on the filter utilized. To address this potential variability, all four detectors could be implemented with variable filters to assess whether the number of detected SWRs changes with different filter specifications. Establishing a proposed fixed filter specification for future methodologies could be instrumental in developing a gold standard for an automatic ripple identification approach. Adopting a standardized filter setting can enhance consistency and comparability across different studies, facilitating advancements in SWR identification. Exploring this direction could improve the accuracy and reliability of SWR detection methodologies and contribute to the establishment of a robust and widely accepted automatic ripple identification methodology.

Further analysis of the existing ripple identification methodologies can shed light on the criteria that lead each detector to reject false-negative events. For instance, the Energy-based detector utilizes a criterion that requires at least six peaks to be above the threshold. However, we found instances where it rejected a true SWR with only five peaks surpassing the threshold. Similarly, the Multi-statistical detector employs six criteria, resulting in the algorithm detecting the fewest events. Eliminating criteria such as the Fano factor, which was found to reject a true positive event, could enhance the sensitivity of this detector without compromising its specificity. By identifying the specific criteria that significantly impact each detector's performance, we can propose improvements to these algorithms. Understanding how individual criteria affect the detection process can lead to the development of enhanced and more accurate ripple identification methodologies. This analysis could help refine the existing detectors and pave the way for implementing improved algorithms that better capture true SWRs while minimizing the risk of false-negative events.

Based on the alcohol administration model and the methodologies implemented in this study, no consistent group-level differences were observed between the sham, vehicle, and alcohol groups in the occurrence rate, duration, or peak frequency of SWRs across all conditions. A significant difference in SWR peak frequency was detected among groups prior to the open field test (*p* = 0.003), whereas no significant differences were found for the T-labyrinth task (*p* = 0.451). This suggests that the short-term alcohol exposure used here was insufficient to induce stable neurophysiological alterations typically associated with chronic alcohol use (Egervari et al., 2021; Santín et al., 2000; Zahr, 2017). However, the significant increase in SWR peak frequency within the experimental group after the open field test indicates a possible transient, task-related compensatory response, rather than a lasting modification of hippocampal circuitry. This interpretation remains speculative, as no direct evidence of compensatory mechanisms was assessed. Overall, these findings highlight the need for longer and more robust chronic alcohol models to evaluate potential long-term effects on hippocampal function, SWR generation, and memory-related circuitry. Further studies will be essential to determine whether extended alcohol exposure can produce the structural and functional alterations predicted by prior literature.

While the transient compensation mechanisms may explain the observed results in the short term, it remains essential to investigate the lasting effects of alcohol on neural activity and memory consolidation processes. Further studies with extended periods of alcohol exposure and a stable pathological state could provide valuable insights into the persistent alterations in brain structures and SWR generation.

## Data Availability

The raw data supporting the conclusions of this article will be made available by the authors, without undue reservation.
